# Effect of Dietary Amylose/Amylopectin Ratio on Intestinal Health and Cecal Microbes’ Profiles of Weaned Pigs Undergoing Feed Transition or Challenged With *Escherichia coli* Lipopolysaccharide

**DOI:** 10.3389/fmicb.2021.693839

**Published:** 2021-07-20

**Authors:** Can Yang, Min Wang, XiaoWu Tang, HuanSheng Yang, FengNa Li, YanCan Wang, Jianzhong Li, YuLong Yin

**Affiliations:** ^1^Hunan International Joint Laboratory of Animal Intestinal Ecology and Health, Laboratory of Animal Nutrition and Hunan Health, College of Life Science, Hunan Normal University, Changsha, China; ^2^Hunan Provincial Key Laboratory of Biological Resources Protection and Utilization in Nanyue Mountain Area, College of Life Sciences and Environment, Hengyang Normal University, Hengyang, China; ^3^Key Laboratory of Agro-ecological Processes in Subtropical Region, Hunan Provincial Engineering Research Center of Health Livestock, Institute of Subtropical Agriculture, Chinese Academy of Sciences, Changsha, China; ^4^College of Bioengineering, Hunan Vocational Technical College of Environment and Biology, Hengyang, China

**Keywords:** dietary amylose/amylopectin ratio, diarrhea, microorganism, metabolites, weaned piglet

## Abstract

**Background:**

Dietary amylose/amylopectin ratio (DAR) plays an important role in piglets’ intestinal health. It is controversial whether diarrhea could be relieved by changing DAR in weaning piglets.

**Methods:**

Sixty (Landrace × Yorkshire) castrated male pigs (initial body weight (BW) 6.51 ± 0.64 kg) were randomly allocated to five groups (one pig per cage and 12 replicates per group) according to their BW. Piglets received diets with different DARs (0.00, 0.20, 0.40, 0.60, and 0.80) for 29 days. Feed transition occurs at day 15. The piglets were challenged with lipopolysaccharides (*Escherichia coli* LPS, 100 μg/kg BW) on day 29 by intraperitoneal injection at 12 h before slaughter. Chyme was collected for pH value, short-chain fatty acid (SCFA), and cecal microbe analysis using 16S rRNA gene sequencing; mucosa was sampled for detecting gene expression.

**Results:**

Rate and degree of diarrhea were higher when DAR was 0.40 than when it was 0.20 and 0.80 during the third week (*P* < 0.05). The chyme pH value in the cecum was higher (*P* < 0.05) in 0.20 DAR than in 0.00 and 0.80 DARs, but with no significant difference compared with 0.40 and 0.60 DARs (*P* > 0.05). Cecal isobutyric acid and isovaleric acid concentrations were higher in 0.20 than in other groups (*P* < 0.01). Cecal SCFAs such as acetic acid, propionic acid, and total SCFA, concentrations were higher in 0.40 DAR than in 0.00, 0.60, and 0.80 DARs (*P* < 0.05), but with no significant difference when compared with 0.20 (*P* > 0.05). Cecal crypt depth was lower (*P* < 0.05) in 0.80 than in other groups, but not 0.40. Claudin mRNA expression in the mucosa of the ileum was higher in 0.20 than in other groups (*P* < 0.01). The alpha diversity of cecal microbe representative by goods coverage was higher in group 0.40 when compared with group 0.20 (*P* < 0.05). At the genus level, the abundances of the *Ruminococcaceae_NK4A214_group* and *Anaerotruncus* were higher but that of *Cetobacterium* was lower in the cecal chyme of group 0.20 than that of group 0.60 (*P* < 0.05), with no significant difference compared with other groups (*P* > 0.05). The diarrhea rate during the third week was negatively correlated with the abundances of *Rikenellaceae_RC9_gut_group* and *X.Eubacterium_coprostanoligenes_group* (*P* < 0.05).

**Conclusion:**

Compared with diet high in amylose or amylopectin, diet with DAR 0.40 showed a worse degree of diarrhea in weaned piglets during feed transition. But the intestinal health will be improved the week after the microbes and metabolites are regulated by DAR.

## Implications

Starch is composed of amylose and amylopectin. Our study confirmed that diarrhea was even worse in weaned piglets during the feed transition period when they received diet whose dietary amylose/amylopectin ratio was 0.40. Supplementation of amylose or amylopectin would be beneficial for weaned piglets undergoing feed transition and under lipopolysaccharide stress.

## Background

Weaning is a crucial stage as piglets have to face pathogenic challenges because pathways referring to innate system response were changed during this stage (Bomba et al., 2014). During weaning, piglets are exposed to a number of stressors: abrupt change in diet from milk to solid feed, radical change in the environment, and an immature digestive and immune system. Diarrhea is a consequence of this situation, which accounts for 17% death of piglets born in Europe (Lallès et al., 2007).

Gut microbiota such as segmented filamentous bacteria and *Candidatus Arthromitus* protect against pathogens by regulating the mammalian immune system (Schnupf et al., 2017). Gut microbial composition could be a cause or result of diarrhea; compared with that in healthy piglets, the abundances of Prevotellaceae, Lachnospiraceae, Ruminococcaceae, and Lactobacillaceae were lower in diarrheic piglets (Dou et al., 2017). The gut microbes exert a primordial role in promoting fermentation of fermentable fiber for production of short-chain fatty acid (SCFA). Production of SCFA is dependent on microbial composition and fermentable substrate. Rats with chronic kidney disease received a diet rich in fermentable fiber, promoting the integrity of the intestinal epithelial barrier and then attenuating both local and systemic inflammation (Vaziri et al., 2014). Resistant starch (RS), which cannot be digested in the small intestine but can act as a substrate for microbial fermentation in the large bowel, has been considered to reduce inflammation (Moraes et al., 2016). Dietary amylose/amylopectin ratio (DAR) was positively correlated with RS content, digestibility of starch, and gross energy, which was greater in low-DAR than in high-DAR samples (Li et al., 2015). Numbers of *Bacillus* in the intestine increased after piglets received pea starch diet with a DAR of 0.52 (Han et al., 2012). But the number of total bacteria in the colonic digesta decreased and proinflammatory cytokine interleukin (IL)-1β gene expression increased in growing–finishing pigs which received raw potato starch diet (133.5 g RS/kg diet) (Sun et al., 2015).

*Escherichia coli* lipopolysaccharide (LPS) is commonly used to activate the immune system; the effective dose of LPS was 25–150 mg/kg body weight (BW) of treated pigs (Wang et al., 2011). Gut injury was induced by 100 μg/kg dosage of *E. coli* LPS in pigs (Liu et al., 2008); ileal tumor necrosis factor-α (TNFα) and IL-1β were upregulated 96 h post challenge, which indicated the immune activation of pigs under LPS challenge (Yin et al., 2017). Therefore, the aim of this study was to determine the DAR on intestinal health reflected by inflammation gene expression and intestinal morphological structure of weaned piglets undergoing feed transition or challenged with *E. coli* LPS, and then the gut microbes and its metabolites (SCFA) were analyzed to find out how DAR affects the intestinal health of weaned piglets.

## Materials and Methods

The experimental procedure was reviewed and approved by the Animal Care and Use Committee of the Hunan International Joint Laboratory of Animal Intestinal Ecology and Health, Hunan Normal University.

### Animals and Diets

Sixty 21-day-old castrated male pigs (Landrace × Yorkshire) with an initial average BW of 6.51 ± 0.64 kg were selected, blocked by BW and allotted to five dietary treatments with 12 cages per treatment and one pig per metabolic cage. The experimental diet was formulated according to the nutrient requirements of the National Research Council (National Research Council, 2012) for 7–11 kg pigs. The A, B, C, D, and E diets differed only in DAR, 0.00, 0.20, 0.40, 0.60, and 0.80, respectively (Table 1). DAR was formulated by using different ratios of waxy corn starch (Fuyang Biological Starch Co. Ltd, Dezhou, Shandong, China) and High-Maize 1043 (National Starch and Chemical Company, Shanghai, China). Nursing diets were provided from days 1 to 14; weaned diets were provided from days 15 to 29. Feed and water were provided *ad libitum*. Six pigs from each treatment group were challenged with 100 μg/kg BW LPS (L2880, from *E. coli* O55:B5, Sigma Chemical Inc., St Louis, MO, United States) on day 29 of the experiment by intraperitoneal injection at 12 h before slaughter; sterile saline was administered to six other pigs.

**TABLE 1:** Composition of experimental diets (as-fed basis).

**Ingredients^a^,%**	**Pre-care period**					**Late-care period**				
	0.00	0.20	0.40	0.60	0.80	0.00	0.20	0.40	0.60	0.80
Waxy corn starch	54.80	45.21	38.36	33.43	29.32	53.54	44.17	37.48	32.66	28.64
Hi-maize 1,043	−	9.59	16.44	21.37	25.48	−	9.37	16.06	20.88	24.90
Soybean meal	9.00	9.00	9.00	9.00	9.00	12.00	12.00	12.00	12.00	12.00
Full-fat extruded soybean	9.00	9.00	9.00	9.00	9.00	10.00	10.00	10.00	10.00	10.00
Fermented soybean meal	8.00	8.00	8.00	8.00	8.00	10.00	10.00	10.00	10.00	10.00
Fish meal	5.00	5.00	5.00	5.00	5.00	5.80	5.80	5.80	5.80	5.80
Whey, dried	5.00	5.00	5.00	5.00	5.00	5.00	5.00	5.00	5.00	5.00
Plasma protein powder	4.84	4.84	4.84	4.84	4.84	−	−	−	−	−
Monocalcium phosphate	1.17	1.17	1.17	1.17	1.17	1.33	1.33	1.33	1.33	1.33
Soybean oil	1.00	1.00	1.00	1.00	1.00	−	−	−	−	−
Premix^*b*^	0.92	0.92	0.92	0.92	0.92	0.92	0.92	0.92	0.92	0.92
Choline chloride	0.08	0.08	0.08	0.08	0.08	0.08	0.08	0.08	0.08	0.08
Limestone	0.74	0.74	0.74	0.74	0.74	0.72	0.72	0.72	0.72	0.72
DL-Lysine	0.23	0.23	0.23	0.23	0.23	0.21	0.21	0.21	0.21	0.21
DL-Methionine	0.22	0.22	0.22	0.22	0.22	0.21	0.21	0.21	0.21	0.21
Salt	−	−	−	−	−	0.19	0.19	0.19	0.19	0.19
Total	100	100	100	100	100	100	100	100	100	100
Calculated nutrient content^c^										
Amylose/amylopectin ratio^d^	0.00	0.20	0.40	0.60	0.80	0.00	0.20	0.40	0.60	0.80
Digestive energy, kcal/kg	3,500	3,500	3,500	3,500	3,500	3,408	3,408	3,408	3,408	3,408
Crude protein,%	18.50	18.50	18.50	18.50	18.50	18.00	18.00	18.00	18.00	18.00
Ca,%	0.85	0.85	0.85	0.85	0.85	0.92	0.92	0.92	0.92	0.92
Av.P,%	0.42	0.42	0.42	0.42	0.42	0.48	0.48	0.48	0.48	0.48
Salt,%	0.50	0.50	0.50	0.50	0.50	0.50	0.50	0.50	0.50	0.50
Amino acids,%SID^e^										
Lys	1.45	1.45	1.45	1.45	1.45	1.31	1.31	1.31	1.31	1.31
TSAA	0.79	0.79	0.79	0.79	0.79	0.71	0.71	0.71	0.71	0.71
Thr	0.83	0.83	0.83	0.83	0.83	0.74	0.74	0.74	0.74	0.74
Trp	0.25	0.25	0.25	0.25	0.25	0.23	0.23	0.23	0.23	0.23

*^a^DAR of waxy corn starch and Hi-maize 1,043 were 0.00 and 1.00, respectively.*

*^b^Vitamin–mineral premix supplied per kilogram of feed: 10,000 IU of vitamin A, 1,000 IU of vitamin D3, 80 IU of vitamin E, 2.0 mg of vitamin K3, 0.03 mg of vitamin B12, 12 mg of riboflavin, 40 mg of niacin, 25 mg of D-pantothenic acid, 0.25 mg of biotin, 1.6 mg of folic acid, 3.0 mg of thiamine, 2.25 mg of pyridoxine, 300 mg of choline chloride, 150 mg of Fe (FeSO_4_), 100 mg of Zn (ZnSO_4_), 30 mg of Mn (MnSO_4_), 25 mg of Cu (CuSO_4_), 0.5 mg of I (KIO_3_), 0.3 mg of Co (CoSO_4_), 0.3 mg of Se (Na_2_SeO_3_), and 4.0 mg of ethoxyquin.*

*^c^Nutrient content of diets based on estimated nutrient contents of ingredients according to NRC (2012).*

*^d^Analyzed data. Amylose and amylopectin contents were determined by using their assay kits (l-AMYL, Megazyme International Ireland Ltd., Wicklow, Ireland).*

*^e^SID, standardized ileal digestible.*

### Slaughter Surveys and Sampling

Diarrhea of piglets was recorded every day during the experimental period. On day 29, 12 h post challenge, pigs were slaughtered via electrical stunning followed by exsanguination. Digesta were collected from the stomach, proximal duodenum, distal jejunum, end of ileum, cecum, and colon. Mucosa from the jejunum and ileum was sampled by scraping the intestinal wall using a glass slide. Mucosa and digesta samples were stored at −80°C for further analysis. Intestinal segments such as the cecum and colon were fixed by immersion in 10% buffer neutral formalin.

## Analysis

### Diarrhea Incidence

Fecal consistency was scored as follows: 0 = normal to 5 = liquid. The diarrhea degree was the sum of the fecal scores for every piglet each week. The diarrhea rate was calculated using following formula: diarrhea rate = total number of pigs with diarrhea/(total number of pigs × experimental days) × 100. The total number of pigs with diarrhea referred to the number of pigs with diarrhea observed on each day.

### pH Value Test

The pH value of digesta was measured using a pH meter (Testo 206, pH meter, Testo AG, Lenzkirch, Germany).

### Intestinal Morphology

Fixed intestinal tissue samples were dehydrated, embedded, sectioned, and stained with hematoxylin and eosin. Mean crypt depth was measured using × 40 combined magnification and an image processing and anastem software (Leica Imaging Systems Ltd., Cambridge, United Kingdom). A minimum of 20 crypts was randomly chosen and measured per subject. Crypt depth was measured using the IPP software (Media Cybernetics Corporation, United States).

### Volatile Fatty Acid (VFA) Analysis

SCFA of metaphosphoric acid-derived samples was tested according to the method described by Mathew et al. (1996). Gas chromatography (Agilent Technologies 7890B GC System; Agilent) and a DB-FFAP column (30 m × 250 μm × 0.25 μm) were used to determine propionate, acetate, butyrate, valerate, isobutyric, and isovaleric acid concentrations.

### Gene Expression Analysis by RT-qPCR

Total RNA was isolated from mucosa using RNAiso Plus (Takara, Dalian, China); reverse-transcription reactions were conducted using an RT reagent kit (Takara, Dalian, China). Quantity and quality of RNA were determined with the NanoDrop ND-2000 spectrophotometer system (Thermo Fisher Scientific Inc., Wilmington, DE, United States). Real-time (RT)-PCR primers related to tight junction proteins (claudin, ZO-1, or occludin), inflammation cytokines (TNFα and IL-1β), and 18S gene were designed (Table 2). RT-PCRs were performed on a MyIQ instrument (Bio-Rad, Hercules, California, United States) using a SYBR Green quantitative PCR mix (Takara, Dalian, China).

**TABLE 2:** RT-PCR primers related to tight junction and inflammation.

**Name^1^**	**F/R**	**Primer**	**GenBank accession number**	**Productive size (bp)**
Claudin-1	F	TTTCCTCAATACAGGAGGGAAGC	NM_001244539.1	196
	R	CCCTCTCCCCACATTCGAG		
Occludin-1	F	CAGGTGCACCCTCCAGATTG	NM_001163647.2	176
	R	GGACTTTCAAGAGGCCTGGAT		
ZO-1	F	CTGAGGGAATTGGGCAGGAA	XM_003353439.2	169
	R	TCACCAAAGGACTCAGCAGG		
IL-1β	F	ACCTGGACCTTGGTTCTC	NM_214055.1	85
	R	GGATTCTTCATCGGCTTC		
TNFα	F	ACGCTCTTCTGCCTACTGC	EU682384.1	128
	R	TCCCTCGGCTTTGACATT		
18S	F	GAGCGAAAGCATTTGCCAAG	NM_001206359.1	140
	R	GGCATCGTTTATGGTCGGAAC		

*^1^*ZO-1, tight junction protein zonula occluden-1; IL-1 β, interleukin 1β; TNFα, tumor necrosis factor alpha.**

### 16S Ribosomal RNA Sequencing

Total genome DNA from cecal digesta was extracted by using a DNA Isolation Kit (MoBio Laboratories, Carlsbad, CA, United States). 16S rRNA sequencing was conducted in a testing institution (Novogene, Beijing, China). Pyrosequencing was done on the V3–V4 variable region of the bacterial 16S rRNA genes. PCR amplification was conducted using the barcoded universal bacterial primers. Samples in triplicate were pooled for sequencing on the HiSeq 2500 platform (Illumina, United States) (Yin et al., 2018). Paired-end sequences were generated and analyzed using QIIME software (version 1.9.1). Sequences were quality checked, with a threshold of 97% set to assign reads to operational taxonomic units (OTUs). The Greengenes database was used as a reference for taxonomy assignment. Bacterial analysis was conducted using R software (version 2.15.3) with the “vegan” package.

### Statistical Analysis

Gene expression data were analyzed using the method of Livak and Schmittgen (2001). Data of diarrhea occurrence were examined by a single-factor design. The cecal microbial community was examined by a single-factor design, and the false discovery rate (*Q*-value) method (Benjamini and Hochberg, 1995) was used to correct *P*-values. Other data were analyzed considering DAR and LPS stress as main effects as well as the interaction between DAR and LPS stress. Analysis of variance was conducted on SAS 8.0 (SAS Institute Inc., Cary, NC, United States) using the general linear model (GLM) procedure. Differences between groups were analyzed using Student’s *t*-test. Results were presented as least squares means ± standard error. Means were considered statistically different at *P* < 0.05 and highly significant at *P* < 0.01.

## Results

### Diarrhea Occurrence

The diarrhea rate and diarrhea degree were higher in 0.40 DAR than in 0.20 and 0.80 DARs during the third week (*P* < 0.05). No statistical difference of diarrhea rate and degree could be observed between different experimental treatments during the first, second, and fourth weeks and at the total of 4 weeks (*P* > 0.05) (Table 3).

**TABLE 3:** Effect of DAR on diarrhea occurrence of weaned piglets challenged with *E. coli* LPS.

**Items**	**0.00**	**0.20**	**0.40**	**0.60**	**0.80**	**SEM**	***P*-value**
**Diarrhea rate, head days**							
First week	19.64	17.86	9.52	11.90	13.69	2.08	0.52
Second week	13.89	24.31	29.86	21.53	20.14	2.76	0.46
Third week	23.21^AB^	11.31^BC^	29.17^A^	18.45^ABC^	7.74^C^	2.25	0.02
Fourth week^1^	19.64	8.33	24.40	19.05	9.52	2.79	0.29
Total 4 weeks^1^	19.29	15.12	22.99	17.59	12.50	1.51	0.24
**Diarrhea degree, score**							
First week	7.42	6.75	4.00	5.67	5.75	0.90	0.79
Second week	5.83	10.42	13.25	10.50	8.33	1.31	0.47
Third week	10.42^AB^	4.67^B^	14.25^A^	9.17^AB^	3.08^B^	1.19	0.03
Fourth week^1^	8.92	3.67	11.92	10.17	4.42	1.45	0.30
Total 4 weeks^1^	32.58	25.50	43.42	35.50	21.58	3.19	0.22

*^1^Statistical analysis ended on day 27. Means within each row without the same superscript letter significantly differ (P < 0.05).*

### pH Value and VFA of Digesta

No significant difference of pH was observed between five groups in digesta of the stomach, jejunum, and ileum (*P* > 0.05) (Table 4). Cecal pH was affected by DAR and LPS stress, and pH value was higher in 0.20 DAR than in 0.80 and 0.00 DARs (*P* < 0.05), but with no significant difference when compared with 0.40 and 0.60 DARs (*P* > 0.05). Cecal (not 0.80 DAR) and colonic (not 0.00 DAR) pH values increased after LPS stress (*P* < 0.05). No effect of interaction between DAR and LPS stress was found on pH value (*P* > 0.05).

**TABLE 4:** Effect of DAR on the pH value of the digesta of weaned piglets challenged with *E. coli* LPS.

**Items^1^**	**0.00**		**0.20**		**0.40**		**0.60**		**0.80**		**SEM**	***P*-value**		
	**LPS**	**SAL**	**LPS**	**SAL**	**LPS**	**SAL**	**LPS**	**SAL**	**LPS**	**SAL**		**DAR**	**STRESS**	D*S
Stomach	2.77	3.15	3.40	3.46	3.54	3.22	3.79	2.62	3.39	2.79	0.11	0.91	0.35	0.69
Jejunum	5.85	5.57	5.76	6.35	6.14	5.79	5.54	6.16	5.28	6.13	0.08	0.92	0.33	0.57
Ileum	6.84	6.50	6.21	7.17	6.49	7.20	6.92	6.66	6.84	7.10	0.05	0.83	0.15	0.11
Cecum	6.14^B^	6.13	6.71^A^	6.34	6.43BA	6.15	6.55BA	6.06	6.03^B^	6.10	0.04	0.02	0.02	0.22
Colon	6.55	6.76	6.80	6.56	6.92	6.32	6.93	6.37	6.91	6.40	0.05	1.00	0.003	0.14

*^1^LPS, lipopolysaccharide; SAL, saline; DAR, dietary amylose/amylopectin ratio.*

*Means within each row without the same superscript letter significantly differ (P < 0.05).*

The DAR had no significant effect on SCFA such as acetic acid, propionic acid, isobutyric acid, butyric acid, isovaleric acid, and total amount of SCFA concentration in the jejunum (*P* > 0.05) (Table 5). Jejunal butyric acid tended to increase after LPS stress (*P* = 0.06) but not in 0.60 DAR. Valeric acid concentration in the jejunum increased after LPS stress in groups with 0.00, 0.20, and 0.80 DARs but decreased in groups with 0.40 and 0.60 DARs (*P* < 0.05).

**TABLE 5:** Effect of DAR on concentrations of VFAs in the digesta of weaned piglets challenged with *E. coli* LPS.

**Items^1^, μg/g wet sample**	**0.00**		**0.20**		**0.40**		**0.60**		**0.80**		**SEM**	***P*-value**		
	**LPS**	**SAL**	**LPS**	**SAL**	**LPS**	**SAL**	**LPS**	**SAL**	**LPS**	**SAL**		**DAR**	**STRESS**	D*S
**Jejunum**														
Acetic acid	41.84	30.39	300.98	56.52	58.57	17.84	69.97	99.37	49.07	27.86	10.63	0.24	0.28	0.33
Propionic acid	11.14	7.23	77.46	28.46	22.61	7.96	13.32	20.08	24.19	10.67	2.95	0.28	0.33	0.70
Isobutyric acid	0.00	0.00	12.42	0.00	0.00	0.00	0.00	3.88	0.00	0.00	0.45	0.55	0.59	0.83
Butyric acid	11.14	7.23	21.90	6.24	22.61	7.96	13.32	20.08	24.19	10.69	1.97	0.78	0.06	0.40
Isovaleric acid	6.68	7.69	20.16	0.00	0.00	4.64	9.11	25.37	15.59	13.49	1.84	0.38	0.81	0.28
Valeric acid	27.52	9.05	46.52	0.00	27.48	33.69	7.90	18.08	37.25	23.59	1.88	0.23	0.04	0.004
Total amount of SCFA	97.53	54.37	520.04	84.98	108.66	64.13	100.30	173.35	163.97	162.40	13.92	0.25	0.26	0.13
**Ileum**														
Acetic acid	594.22	927.69	786.89	631.66	541.72	928.44	634.98	544.22	942.19	848.87	37.54	0.18	0.33	0.08
Propionic acid	44.18	58.87	91.71	41.11	81.28	73.66	48.28	60.25	77.71	104.72	11.91	0.83	0.97	0.88
Isobutyric acid	0.00	0.00	0.00	0.00	0.00	4.25	0.00	0.00	0.00	0.00	0.47	0.55	0.39	0.55
Butyric acid	48.89	52.47	51.66	28.96	44.53	62.24	27.83	30.76	78.89	68.53	5.35	0.14	0.87	0.82
Isovaleric acid	0.00	0.00	0.00	10.03	0.00	17.68	0.00	0.00	0.00	3.04	1.25	0.15	0.02	0.15
Valeric acid	1.32	0.00	0.00	0.00	0.00	0.00	0.00	0.00	0.00	0.00	0.15	0.60	0.39	0.60
Total amount of SCFA	688.62	1,039.03	930.26	711.75	667.53	1,086.28	711.09	635.23	1,098.79	1,025.16	39.02	0.06	0.33	0.06
**Cecal**														
Acetic acid	2,746.25^B^	3,610.85	3,433.77^AB^	4,426.02	4,198.72^A^	4,643.30	3,093.49^B^	3,187.17	2,901.98^B^	3,329.84	148.66	0.03	0.08	0.90
Propionic acid	1,242.81^B^	1,476.83	1,631.64^*AB*^	1,805.10	2,204.71^A^	1,844.70	1,391.95^*B*^	1,414.73	1,473.23^B^	1,279.86	69.77	0.02	0.87	0.67
Isobutyric acid	86.89^*BCb*^	89.75	211.57^Aa^	135.97	124.75^Bb^	117.36	132.88^BCb^	63.96	82.11^Cb^	70.14	5.42	< 0.0001	0.01	0.10
Butyric acid	585.71	965.03	637.10	712.82	820.75	966.94	802.65	778.01	668.31	758.83	45.61	0.65	0.17	0.75
Isovaleric acid	113.68^Bb^	110.53	309.97^Aa^	184.45	201.58^Bab^	122.02	255.10^Bab^	80.98	117.09^Bb^	83.84	10.09	0.001	0.0003	0.10
Valeric acid	192.85^*B*^	200.68	379.11^A^	292.34	295.05^AB^	232.47	245.74^B^	142.09	211.27^B^	159.77	14.57	0.02	0.06	0.82
Total amount of SCFA	4,968.18^B^	6,453.67	6,603.15^AB^	7,556.70	7,845.56^A^	7,926.79	5,921.81^B^	5,666.94	5,453.97^B^	5,682.28	260.26	0.03	0.37	0.86
**Colon**														
Acetic acid	2,862.32	3,032.80	2,292.78	3,213.04	2,378.01	3,367.35	2,104.43	3,360.75	1,701.21	3,347.96	65.92	0.40	< 0.0001	0.04
Propionic acid	1,225.44	1,169.05	1,080.91	1,280.66	1,030.95	1,375.93	940.75	1,397.74	772.60	1,348.47	33.87	0.71	0.0001	0.09
Isobutyric acid	126.31	132.60	185.56	144.87	161.69	110.50	134.54	128.63	161.95	125.40	5.36	0.26	0.03	0.45
Butyric acid	607.55	750.58	561.84	758.93	685.08	923.08	475.83	861.94	488.16	918.83	31.77	0.60	0.0001	0.62
Isovaleric acid	201.67	200.77	343.89	216.31	273.89	148.26	220.49	186.95	298.49	173.75	11.99	0.24	0.002	0.36
Valeric acid	229.56	225.36	355.00	240.91	259.62	241.07	190.01	236.07	235.32	241.87	9.30	0.08	0.39	0.12
Total amount of SCFA	5,252.84	5,511.16	4,819.99	5,854.72	4,789.24	6,166.18	4,066.05	6,172.07	3,657.73	6,156.28	124.10	0.64	< 0.0001	0.09

*^1^LPS, lipopolysaccharide; SAL, saline; DAR, dietary amylose/amylopectin ratio.*

*Means within each row without the same superscript letter (A, B, and C) significantly differ (P < 0.05) and (a–c) highly significantly differ (P < 0.01).*

Ileal acetic acid, propionic acid, butyric acid, isobutyric acid, valeric acid, and total amount of SCFA were not affected by DAR or LPS stress, except that isovaleric acid of the ileum decreased after LPS stress (*P* < 0.05).

Cecal SCFAs, except butyric acid, were affected by DAR (*P* < 0.05). Acetic acid, propionic acid, and total SCFA concentrations were higher in the group with 0.40 DAR that in groups with 0.00, 0.60, and 0.80 DARs (*P* < 0.05). Isobutyric acid and isovaleric acid concentrations increased after LPS stress (*P* < 0.05) and were higher in the 0.20 DAR group than in the other groups (*P* < 0.05). Valeric acid concentration was higher in the group with 0.20 DAR that in groups with 0.00, 0.60, and 0.80 DARs (*P* < 0.05).

Colonic SCFAs, except valeric acid, were affected by LPS stress but not DAR; the SCFA including acetic acid, propionic acid (not in 0.00), and butyric acid decreased after LPS stress (*P* < 0.05).

### Crypt Depth of the Large Intestine

The crypt depth of the cecum was lower in the 0.80 DAR group than in other groups, except 0.40 DAR (*P* < 0.05). The crypt depth of the colon was not affected by DAR and LPS stress (*P* > 0.05) (Table 6).

**TABLE 6:** Effect of DAR on crypt depth of the intestine of weaned piglets challenged with *E. coli* LPS.

**Items^1^, μm**	**0.00**		**0.20**		**0.40**		**0.60**		**0.80**		**SEM**	***P*-value**		
	**LPS**	**SAL**	**LPS**	**SAL**	**LPS**	**SAL**	**LPS**	**SAL**	**LPS**	**SAL**		**DAR**	**STRESS**	D*S
Cecal	429.98^Aa^	424.59	401.36^Aa^	429.59	396.38^ABab^	385.18	396.34^Aab^	414.19	372.74^Bb^	362.18	5.26	0.01	0.73	0.69
Colon	436.03	475.67	470.36	461.69	450.36	474.08	446.81	477.60	464.20	434.81	7.12	0.96	0.45	0.55

*^1^LPS, lipopolysaccharide; SAL, saline; DAR, dietary amylose/amylopectin ratio.*

*Means within each row without the same superscript letter (A, B, and C) significantly differ (P < 0.05) and (a, b, and c) highly significantly differ (P < 0.01).*

### Expression of Genes Related to Gut Health

Expressions of genes related to tight junction and inflammation in mucosa are shown in Table 7. DAR did not alter the mRNA expression of ZO-1, IL-1β, and TNFα in the mucosa of the jejunum and did not affect mRNA expression of occludin-1, ZO-1, and IL-1β in mucosa of the ileum (*P* > 0.05). LPS stress caused lower mRNA expression of claudin in jejunal mucosa (*P* < 0.05). Claudin-1 mRNA expression was higher in the 0.60 DAR group than in the other groups (*P* = 0.045) in the jejunum, and it was higher in the 0.20 DAR group than in other groups in the mucosa of the ileum (*P* < 0.01). Ingestion of diet with 0.00 DAR resulted in lower TNFα mRNA levels in ileal mucosa compared with diet with 0.40, 0.60, and 0.80 DARs (*P* < 0.05).

**TABLE 7:** Effect of DAR on gene expression of intestinal mucosa of weaned piglets challenged with *E. coli* LPS.

**Items^1^**	**0.00**		**0.20**		**0.40**		**0.60**		**0.80**		**SEM**	***P*-value**		
	**LPS**	**SAL**	**LPS**	**SAL**	**LPS**	**SAL**	**LPS**	**SAL**	**LPS**	**SAL**		**DAR**	**STRESS**	D*S
**Jejunal mucosa**														
Claudin-1	0.75^*B*^	1.04	0.98^*B*^	1.03	1.01^*B*^	1.02	1.11^A^	1.51	0.79^B^	1.16	0.04	0.045	0.01	0.49
ZO-1	0.92	1.03	0.96	1.04	0.96	1.00	0.82	0.71	0.61	0.96	0.04	0.15	0.22	0.46
IL-1β	0.84	1.11	0.60	0.91	0.39	0.73	0.65	0.77	0.37	0.87	0.08	0.53	0.06	0.97
TNFα	1.25	1.06	0.75	1.63	0.61	0.83	0.65	0.73	0.47	0.79	0.13	0.50	0.32	0.78
**Ileal mucosa**											0.00			
Occludin-1	1.49	1.06	1.27	1.93	1.08	1.44	0.98	1.05	1.08	1.62	0.10	0.55	0.26	0.51
Claudin-1	1.15^Bb^	1.04	2.37^Aa^	2.20	1.25^Bb^	1.53	1.13^Bb^	1.24	1.22^Bb^	1.38	0.05	< 0.0001	0.60	0.60
ZO-1	1.07	1.06	1.07	1.25	1.21	1.02	0.82	0.92	0.90	1.11	0.06	0.63	0.65	0.84
IL-1β	0.80	1.14	1.12	1.60	0.79	1.16	0.62	2.45	1.00	1.16	0.18	0.82	0.09	0.60
TNFα	0.76^Cb^	1.02	0.97^BCab^	1.02	1.96^Aa^	1.25	1.30^Aa^	1.84	1.46^ABab^	1.42	0.07	0.01	0.87	0.10

*^1^LPS, lipopolysaccharide; SAL, saline; DAR, dietary amylose/amylopectin ratio.*

*Means within each row without the same superscript letter (A, B, and C) significantly differ (P < 0.05) and (a, b, and c) highly significantly differ (P < 0.01).*

*ZO-1, tight junction protein zonula occluden-1; IL-1β, interleukin 1β; TNFα, tumor necrosis factor alpha.*

### The Bacterial Community Composition in the Cecum

**FIGURE 1:**
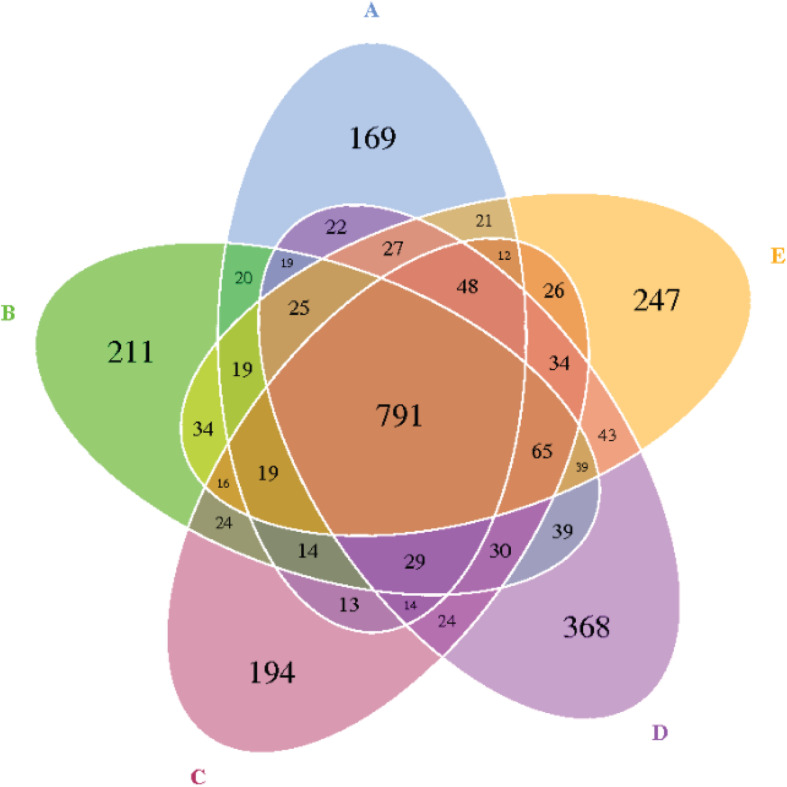
The Venn diagram of the shared and unique OTUs between different DAR groups. DARs of **(A–E)** were 0.00, 0.20, 0.40,0.60, and 0.80 respectively.

**FIGURE 2:**
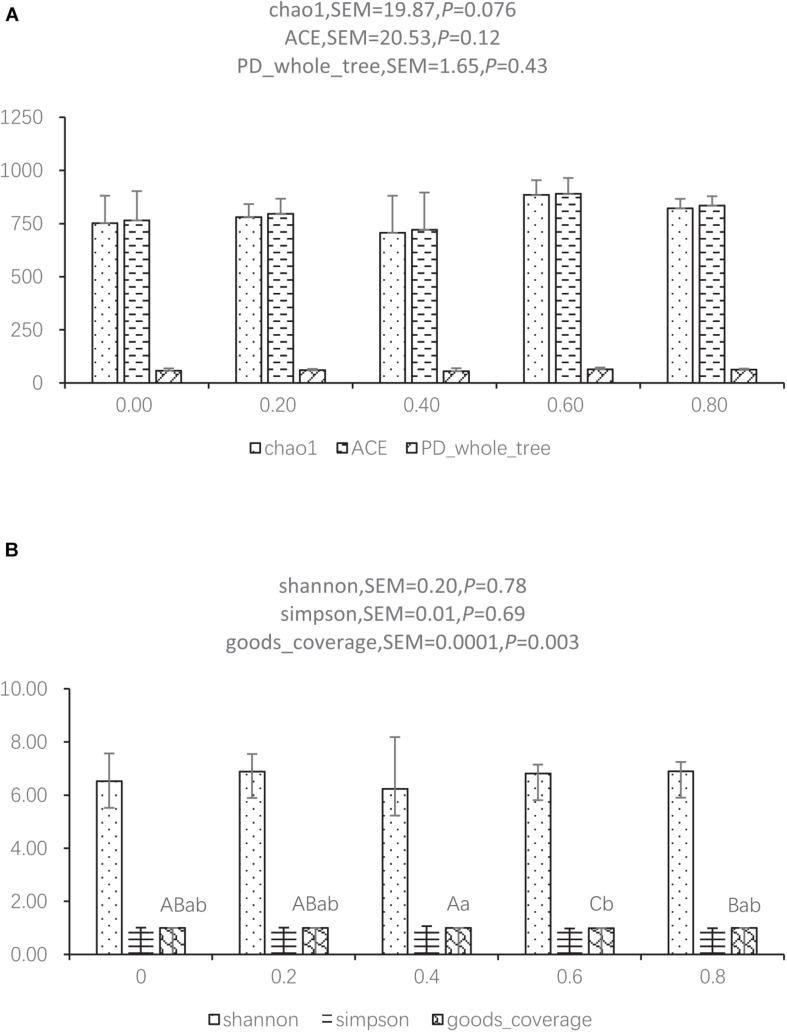
Alpha diversity index between different DAR groups represented by chao1, ACE, PD_whole tree **(A)** and shannon, simpson, and goods coverage **(B)**.

The reads for each sample are in the range of 70,049–96,176. After quality trimming and chimera checking, each sample has 77,296 ± 7,459 tags with a minimum length of 410 nucleotides and a maximum length of 426 nucleotides. Seven hundred and ninety-one OTUs were shared by the five groups, and 169, 211, 194, 368, and 247 OTUs were found only in the ceca of 0.00, 0.20, 0.40, 0.60, and 0.80 DAR groups, respectively (Figure 1). No significant differences were found on Shannon, Simpson, ACE, and PD_whole tree indexes of bacteria between different DAR groups (Figures 2A,B). The alpha diversity of cecal microbes in the 0.40 DAR group represented by chao1 tended to be lower than that in the 0.60 DAR group (*P* = 0.076), with no significant difference compared with other groups (*P* > 0.05). The alpha diversity of cecal microbes in 0.20 and 0.40 DAR groups represented by goods coverage was lower than in the 0.60 DAR group (*P* < 0.05), with no significant difference compared with the other groups (*P* > 0.05).

**FIGURE 3:**
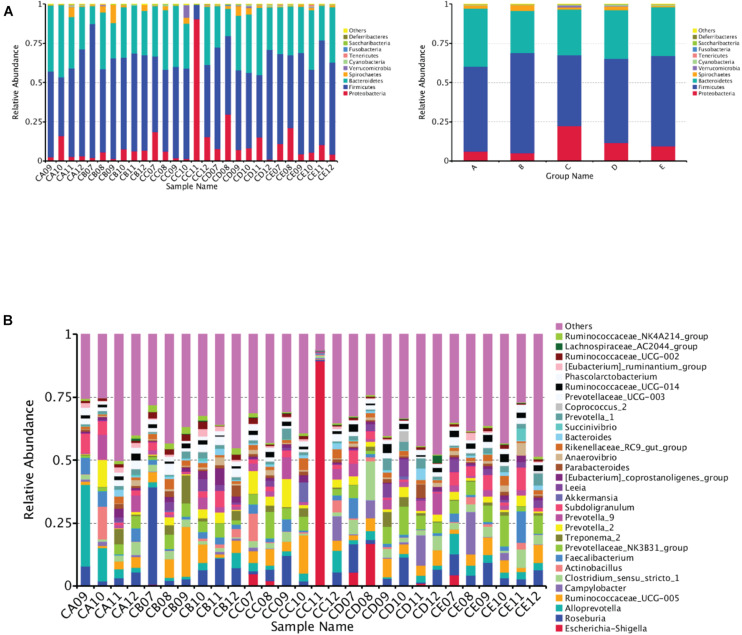
Bar graph showing the top 10 **(A)** or top 30 **(B)** phylum level compositions of bacteria. Color-coded bar plot showing the relative abundance of bacterial phyla across the different samples (**A**, left) or different groups (**A**, right). One representative sequence from a set of related sequences belonging to the same OUT was selected for continuous species annotation with an RDP classifier, and the bacterial composition at the phylum level of each sample was stated and visualized with a histogram. The first C representing it comes from the cecal chyme. The second character represented DAR. DARs of A, B, C, D, and E were 0.00, 0.20, 0.40,0.60, and 0.80, respectively.

At the phylum level, Firmicutes, Bacteroidetes, Proteobacteria, and Spirochaetes were predominantly found in the cecal samples from different DAR groups. No significant difference was found in the phylum between different DAR groups (*P* > 0.05) (Figure 3A). At the genus level, the abundances of the *Ruminococcaceae_NK4A214_group* (*P* < 0.05) and *Anaerotruncus* (*P* < 0.01) in the cecal chyme of the 0.20 DAR group were significantly higher than that in the 0.60 DAR group, with no significant difference compared with other groups (*P* > 0.05) (Figure 3B). The abundance of *Cetobacterium* in the cecal chyme was significantly lower in the 0.20 DAR group than in the 0.60 DAR group (*P* < 0.01), with no significant difference compared with other groups (*P* > 0.05).

### Relationship Between Bacterial Abundance and Apparent Indicators

**FIGURE 4:**
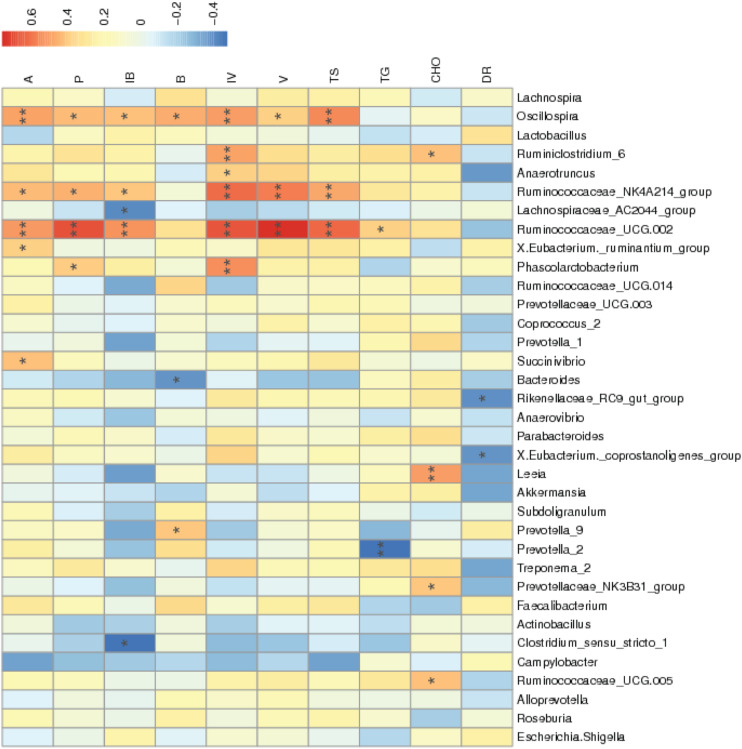
Correlation of the environment and abundance of bacteria at the genus level. Species information is arranged in rows, and environment factors are arranged vertically and are on the horizontal axis (*x*-axis). Different colors indicate the relative abundance between species and environment factors; *r* < 0 represented a negative correlation, and *r* > 0 represented a positive correlation. *Means *P* < 0.05. **Means *P* < 0.01. A, acetic acid; P, propionic acid; IB, isobutyric acid; B, butyric acid; IV, isovaleric acid; V, valeric acid; TS, total amount of SCFA; TG, triglyceride; CHO, cholesterol; DR, diarrhea rate during the third week.

According to Figure 4, the diarrhea rate during the third week was negatively correlated with the abundances of the *Rikenellaceae_RC9_gut_group* and *X.Eubacterium_coprostanoligenes_group* (*P* < 0.05). The abundances of *Ruminococcaceae_UCG.002* and *Ruminococcaceae_NK4A214_group* were positively correlated with cecal total SCFA, acetic acid, propionic acid, isobutyric acid, isovaleric acid, and valeric acid concentrations (*P* < 0.05). The abundance of *Anaerotruncus* was positively correlated with cecal isovaleric acid concentrations (*P* < 0.05). The abundances of *Ruminococcaceae_UCG.005*, *Prevotellaceae_NK3B31_group*, *Leeia*, and *Ruminiclostridium_6* were positively correlated with serum cholesterol concentrations (*P* < 0.05).

## Discussion

An inappropriate imbalance between pro-inflammatory cytokines and the anti-inflammatory cytokines would lead to inflammation in the bowel. Elevation of TNFα and IL-6 is the hallmark of acute bowel inflammation (Kim et al., 2010). Therefore, mucosal cytokine profiling results suggest that there was acute bowel inflammation in the 0.40 and 0.60 DAR groups but not in 0.00 and 0.20 DAR groups under 100 μg/kg of LPS stress in weaned pigs. This is inconsistent with other reports which showed that rats with chronic kidney disease supplemented with amylopectin exhibited inflammation, activation of NFκB, upregulation of pro-inflammatory cytokines, and disruption of colonic epithelial tight junction, but diet containing high RS could significantly attenuate these abnormalities (Vaziri et al., 2014). RS has proven to be effective in reducing inflammation in the state of the disease (Aliasgharzadeh et al., 2015). A study found a reduction of TNFα concentration in prediabetes patients supplemented with 45 g/day high-amylose maize for 12 weeks (Peterson et al., 2018). Supplementation of HAM-RS2 led to a decrease in serum IL-6 and TNFα in end-stage renal disease patients (Laffin et al., 2019). Moreover, consumption of retrograded high-amylose corn RS at 15% may protect the colon by enhancing anti-inflammatory cytokine IL-10 abundance in pigs, without affecting TNFα and IL-6 abundances in the colon (Fan et al., 2012). Though gut injury occurred in piglets that received 60 μg/kg (Waititu et al., 2016) or 100 μg/kg (Hou et al., 2010) of *E. coli* LPS and injection of LPS stimulated the production of IL-1, TNFα, and interferon (IFN)-γ (Kluger, 1991), but 12 h post challenge, LPS showed no effect on TNFα and IL-1β mRNA expressions in jejunal and ileal mucosa of weaning pigs in our result. The acute bowel inflammation in the 0.40 DAR group was due to severe diarrhea occurring during the third week before LPS stress.

Homeostasis of gut microbiota in the 0.40 DAR group might be disrupted because of the diarrhea. Phylum Proteobacteria abundance was higher in the 0.40 DAR group. A previous study indicated that the abnormal increase of Prevotellaceae abundance could exacerbate the occurrence of inflammation (Elinav et al., 2011). Genus *Sutterella* belongs to the Prevotellaceae phylum, and it has been found elevated in feces of dogs with acute hemorrhagic diarrhea (Suchodolski et al., 2012). The *Rikenellaceae_RC9_gut_group* increased in mice fed with high-fat diet with high-dose genistein (Zhou et al., 2018) and in an isoproterenol-induced acute myocardial ischemia group (Sun et al., 2019). We observed a significant negative correlation between abundance of the *Rikenellaceae_RC9_gut_group* and diarrhea rate during the third week in the present study. Thus, the increase of the *Rikenellaceae_RC9_gut_group* might be associated with gut inflammation. Although piglets from the 0.40 DAR group suffered severe diarrhea, they got the same average daily gain and feed intake as other groups during the whole four experimental weeks (data not shown). This result, in part, might be due to SCFAs’ inflammation-modulating response. Piglets fed a diet with 0.40 DAR showed a significant increase in cecal SCFA compared with those fed a diet with 0.00, 0.60, and 0.80 DARs. SCFAs possess anti-inflammatory characteristics by increasing colonic regulatory T cells (Arpaia et al., 2013) and production of pro-inflammatory cytokines (Freeland and Wolever, 2010). Formation of pro-inflammatory and pro-oxidant uremic toxins from colonic bacteria decreased because of SCFA production increase and intestinal pH reduction (Vaziri et al., 2014).

Piglets fed a 0.20 DAR diet exhibited less microbial diversity than piglets fed a 0.6 DAR diet. More microbial diversity had a relation with a healthier phenotype generally (Human Microbiome Project Consortium, 2012). Intestinal microbiota can maintain the intestinal barrier by affecting intestinal permeability, enhancing the transfer of harmful substances into the blood, and stimulating inflammatory response (Kelly et al., 2016). The increase of gut microbiota such as *Ruminococcaceae_NK4A214_group* and *Anaerotruncus* in 0.20 DAR could result in an increase in cecal SCFA such as isobutyric acid, isovaleric acid, and valeric acid concentrations. Higher levels of iso-branched-chain fatty acids (BCFAs) may be associated with alteration in the metabolism of branched-chain amino acids (BCAAs) such as valine, leucine, and isoleucine, which can serve as precursors of BCFAs (Wallace et al., 2018). Thus, the increased production of isobutyric acid and isovaleric acid should indicate increased protein degradation during LPS stress in the 0.20 DAR group. Both Ile and Leu in the liver were increased after LPS stress in other groups but decreased after LPS in the 0.20 DAR group (data not shown). Plasma urea nitrogen levels increased after LPS challenge because of muscle proteolysis (Webel et al., 1997). There was an inverse correlation between serum iso-BCFAs and inflammatory marker C-reactive proteins in patients suffering from morbid obesity (Mika et al., 2016). Released amino acids resulting from inflammation seem to be channeled to the liver to synthesize proteins and to serve as an energy source (Owusu-Asiedu et al., 2003). LPS challenge caused lower claudin mRNA expression in the jejunal mucosa. As a result, the intestinal barrier function was improved as claudin mRNA expression increased in the 0.20 DAR group compared with other groups.

## Conclusion

In conclusion, intestinal health was affected by DAR, which was characterized as both rate and degree of diarrhea being high in 0.40 DAR when weaned piglets undergo feed transition. Supplementing the diet with amylose can improve intestinal health through modulating gut microbes, increasing cecal acetic acid and propionic acid contents, and decreasing cecal crypt depth when weaned piglets undergo feed transition. Intestinal health was improved as claudin mRNA expression in the mucosa of the ileum increased, and cecal isobutyric acid and isovaleric acid concentrations increased when weaned piglets experiencing LPS stress received amylopectin.

## Data Availability Statement

The data presented in the study are deposited in the SRN, accession number is PRJNA733844.

## Ethics Statement

Experimental procedure in this study was reviewed and approved by the Animal Care and Use Committee of the Hunan International joint laboratory of animal intestinal ecology and health, Hunan Normal University.

## Author Contributions

YLY and HSY organized the experiment and gave some advice on experiment idea. CY conducted the experiment and was a major contributor in writing the manuscript. CY, MW, XWT, and YCW conducted the experimental analysis. JZL and FNL reviewed the manuscript and gave some advice on the experiment idea. All authors read and approved the final manuscript.

## Conflict of Interest

The authors declare that the research was conducted in the absence of any commercial or financial relationships that could be construed as a potential conflict of interest.
